# Links between posterior pituitary activity, psychometric profile and other endocrine abnormalities in anorexia nervosa: a multimodal evaluation

**DOI:** 10.1192/j.eurpsy.2022.399

**Published:** 2022-09-01

**Authors:** S. Doua, R. Jérôme, B. Traverse, M. Zennadi, C. Boutet, A. Hammour, N. Germain, C. Massoubre, B. Galusca

**Affiliations:** 1 CHU Saint Etienne, Endocrinology, Saint Etienne, France; 2 CERMEP, Nuclear Medecine, Lyon, France; 3 CHU Saint Etienne, Radiology, Saint Etienne, France; 4 CHU Saint Etienne, Psychiatry, Saint Etienne, France

**Keywords:** Anorexia nervosa, Copeptin, Oxytocin, Cerebral opioid tone

## Abstract

**Introduction:**

Opioid system activity was found disturbed in several reward circuit areas in restrictive anorexia nervosa (AN) patients but also at the pituitary level. The role of this specific abnormality in AN physiopathology remains unknown.**O**

**Objectives:**

We aimed to evaluate the relationship of upper mentioned AN abnormality with its classical pituitary features and eating behavior traits.

**Methods:**

PET [^11^C] diprenorphin binding potential (BP_ND_) were processed for each pituitary part in three groups of young women: 12 AN, 11 recovered AN patients (ANrec), and 12 Controls. Anterior pituitary hormones and neurohypophysis (NH) 12 points circadian profile including copeptin and oxytocin, psychological scores were evaluated in these subjects as well as in 13 bulimic (BN) patients.

**Results:**

[^11^C] diprenorphin pituitary binding was found to be fully localized in NH. Only AN patients’ NH present lower [^11^C] diprenorphin BP_ND_ than Controls, interpreted as a higher opioid tone. Both AN and ANrec show lower copeptin/24h than in Controls but no difference in oxytocin. BN showed increased copeptin and low oxytocin. In AN patients copeptin inversely correlate with Restrained Eating while oxytocin correlate with the External Eating score. NH [^11^C] diprenorphin BP_ND_ correlated with leptin but not with copeptin or oxytocin.

**Conclusions:**

Neurohypopysis opioid tone in anorexia nervosa seem not to impact the vasopressin or oxytocin release but still may interfere in gonadal axis regulation. Copeptin, a good indicator of hydration state, may be a good tool to detect hidden restrictive or purging behaviors. Specific correlates with AN psychologic features still suggest a physiopathological involvement.

**Disclosure:**

No significant relationships.

